# Emerging Roles of Small GTPases in Islet β-Cell Function

**DOI:** 10.3390/cells10061503

**Published:** 2021-06-15

**Authors:** Rajakrishnan Veluthakal, Debbie C. Thurmond

**Affiliations:** Department of Molecular and Cellular Endocrinology, Arthur Riggs Diabetes & Metabolism Research Institute, City of Hope Beckman Research Institute, Duarte, CA 91010, USA

**Keywords:** small GTPase, actin remodeling, β-cell, guanine nucleotide exchange factors, readily releasable pool, cytoskeleton, diabetes, exocytosis, insulin

## Abstract

Several small guanosine triphosphatases (GTPases) from the Ras protein superfamily regulate glucose-stimulated insulin secretion in the pancreatic islet β-cell. The Rho family GTPases Cdc42 and Rac1 are primarily involved in relaying key signals in several cellular functions, including vesicle trafficking, plasma membrane homeostasis, and cytoskeletal dynamics. They orchestrate specific changes at each spatiotemporal region within the β-cell by coordinating with signal transducers, guanine nucleotide exchange factors (GEFs), GTPase-activating factors (GAPs), and their effectors. The Arf family of small GTPases is involved in vesicular trafficking (exocytosis and endocytosis) and actin cytoskeletal dynamics. Rab-GTPases regulate pre-exocytotic and late endocytic membrane trafficking events in β-cells. Several additional functions for small GTPases include regulating transcription factor activity and mitochondrial dynamics. Importantly, defects in several of these GTPases have been found associated with type 2 diabetes (T2D) etiology. The purpose of this review is to systematically denote the identities and molecular mechanistic steps in the glucose-stimulated insulin secretion pathway that leads to the normal release of insulin. We will also note newly identified defects in these GTPases and their corresponding regulatory factors (e.g., GDP dissociation inhibitors (GDIs), GEFs, and GAPs) in the pancreatic β-cells, which contribute to the dysregulation of metabolism and the development of T2D.

## 1. Introduction

**Figure 1:**
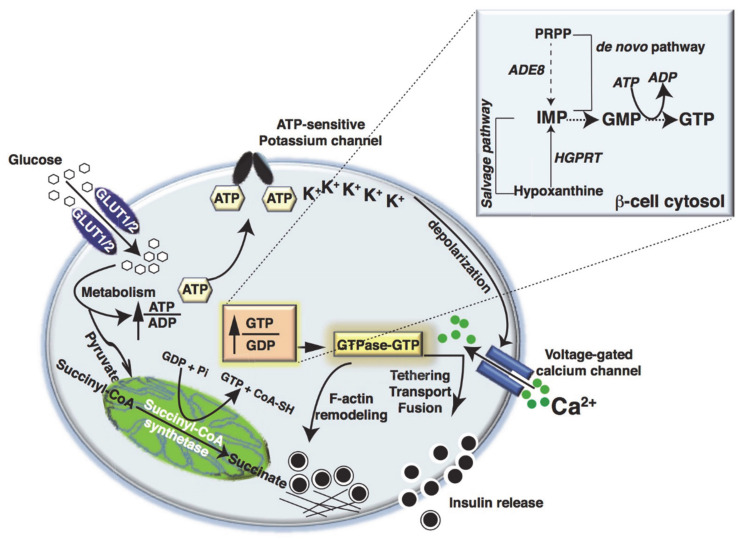
Glucose-stimulated insulin secretion. Glucose is taken up by β-cells via the GLUT1 (human) or GLUT2 (rodent) transporter and undergoes metabolism via mitochondrial oxidation, leading to the generation of ATP. When the ATP/ADP ratio increases, the K*^ATP^* channel closes, and membrane depolarization ensues. Subsequently, voltage-gated calcium channels open, and Ca^2+^ enters to drive insulin granule exocytosis. Meanwhile, in the mitochondria, converion of Succinyl-CoA in the tricarboxylic acid cycle by Succinyl-CoA synthetase causes the formation of mitochondrial GTP. In addition, in the soluble compartment, GTP can be formed from a salvage pathway using hypoxanthine as a substrate. The enzyme hypoxanthine-guanine phosphoribosyltransferase (HGPRT) catalyzes the conversion of hypoxanthine to inosine monophosphate (IMP) and guanine to guanosine monophosphate to GTP. GTP can be also formed by an alternate de novo synthesis pathway, using phosphoribosyl pyrophosphate (PRPP), by the enzyme phosphoribosylglycinamide formyltransferase (ADE8), which then increases the GTP/GDP ratio. This causes differential regulation of GTPases and enhances insulin release.

Pancreatic islet β-cells release the hormone insulin in response to elevated blood glucose concentrations, especially after meals, via a process called glucose-stimulated insulin secretion (GSIS) [1,2]. Insulin maintains whole-body glucose homeostasis by facilitating glucose uptake in primary tissues, such as skeletal muscle and adipose tissue, and reducing gluconeogenesis in the liver [3]. Upon sensing glucose, the β-cells take up glucose via glucose transporters (GLUT1 in humans and GLUT2 in rodents) [4] and initiate glucose metabolism [5,6,7,8]. The metabolism of glucose induces a plethora of signaling events, which change the ATP/ADP ratio, trigger the closure of ATP-sensitive potassium channels, and depolarize the plasma membrane (PM). These actions open PM-localized voltage-dependent Ca^2+^ channels (VDCCs), and the influx of Ca^2+^ from the extracellular space elicits the rapid release of insulin from pre-packaged insulin granules within the β-cell (reviewed elsewhere [9]) (Figure 1). The insulin secretory process is biphasic [10,11,12]; the first phase, lasting 10 min, is associated with rapid and robust insulin secretion, whereas the second phase is associated with a less robust level of secretion but continues as long as elevated glucose levels persist [13]. The Rho, Arf, and Rab families of small GTPases are required for this biphasic pattern of insulin release from the β-cell, overseeing the actin cytoskeletal remodeling, which is required for mobilizing the insulin granules to their docking/fusion sites at the inner PM for regulated release of insulin into the circulation [14,15].

## 2. Metabolic Fate of Glucose in Islet β-Cells

Glucose is converted to pyruvate in the cytosol via glycolysis. However, the levels of cytosolic lactate dehydrogenase are lower in β-cells than in other cell types, and this favors β-cells to continue glycolysis using mitochondrial pyruvate uptake and metabolism [16,17]. This glucose metabolism increases the GTP/GDP ratio in a concentration-dependent manner, and insulin secretion is inversely proportional to GDP levels and directly proportional to GTP/GDP ratios [18,19,20]. Several studies have provided evidence for a particular role of mitochondrial GTP (mtGTP) in GSIS [21,22,23]. For example, the tricarboxylic acid (TCA) cycle enzyme succinyl-CoA synthetase (SCS) catalyzes the substrate-level synthesis of mtGTP and mitochondrial ATP (mtATP) when pyruvate is catabolized in the TCA cycle [22]. Mitochondrial ATP (mtATP) is produced largely by oxidative phosphorylation and is dependent on the mitochondrial membrane potential (ΔΨ). Only a fraction of ATP is directly formed from TCA cycle by the ATP dependent succinyl-CoA synthetase (SCS-ATP). Therefore, changes in the mitochondrial matrix ATP/ADP ratio are limited due to the rapid export of mtATP to the cytosol via the ATP/ADP transporter (AAT). In contrast, mtGTP is only metabolically generated by the GTP-specific isoform of SCS (SCS-GTP). As a result, mtGTP is trapped in the matrix, yielding increases in GTP/GDP that are much more substantial than mtATP [21,22]. Each molecule of glucose metabolized in the β-cell produces approximately one mtGTP, making mtGTP a potentially important fuel signal. In rat islets and the rat clonal β-cell line INS-1 832/13, RNAi suppression of SCS dramatically reduced mtGTP levels and caused a 50% reduction in GSIS [22]. These data suggest a role for mtGTP in governing GSIS via modulation of mitochondrial metabolism, invoking changes in the mitochondrial Ca^2+^ levels.

Conversely, some reports have indicated a small increase in total GTP levels, both in the smaller mtGTP pool and the larger cytosolic GTP pools [18,19,20]. Two pathways are involved in the synthesis of GTP. First, in the salvage pathway, the purine base hypoxanthine is recycled by hypoxanthine-guanine phosphoribosyltransferase (HGPRT) to generate inosine monophosphate (IMP), which serves as a precursor for GMP from which GTP is synthesized. Secondly in the de novo pathway, Phosphoribosyl-glycinamide transformylase (ADE8) catalyzes a step in the de novo purine nucleotide biosynthetic pathway. The purine ring is sequentially constructed from small molecule donors on a ribose 5-phosphate backbone provided by 5-phosphoribosyl-1-pyrophosphate (PRPP) to form the first purine product, IMP. IMP is channeled to form GMP and, subsequently, GTP [24] (Figure 1). Although one molecule of mtGTP is produced by the GTP-specific isoform of SCS per molecule of glucose oxidized, due to slow cytoplasmic exchange, mtGTP is trapped in the matrix; therefore, the increase in GTP/GDP is substantial [25,26]. This regulatory mechanism differs from the regulation of the mitochondrial ATP/ADP ratio, which is limited by the rapid export of mtATP to the cytosol via the ATP/ADP transporter (AAT). Therefore, although the increase in GTP/GDP reflects β-cell TCA cycle activity, the mtATP production rates do not necessarily correlate with the rate of glucose oxidation. The mitochondrial isoform of phosphoenolpyruvate carboxykinase (PEPCK-M) converts oxaloacetate to phosphoenolpyruvate (PEP), driving the co-transport of mtGTP and PEP into the cytosol via the citrate isocitrate carrier (CIC) [27]. In the cytosol, increased mtGTP enhances insulin secretion by increasing the number and/or size of insulin granules, and importantly, promotes the localization of insulin laden granules to the inner surface of the PM, staging them for subsequent release [23]. While mitochondrial GTP may impart resilience to β-cells exposed to glucolipotoxicity-induced metabolic stress [28], the extent to which this can prevent β-cell dysfunction, and progression toward diabetes, remains to be evaluated.

## 3. Metabolic Dysfunction and Small GTPase Signaling in Islet β-Cells

According to the International Diabetes Federation, in the year 2019, approximately 463 million adults (20–79 years of age) were living with diabetes, and it is estimated that by the year 2045, this will rise to 700 million. One in five of the people who are above 65 years old had diabetes, and one in two (232 million) people with diabetes was undiagnosed [29]. β-cell dysfunction contributes toward the etiology of type 2 diabetes (T2D) [30,31]. Undeniably, GSIS from T2D human islets is significantly reduced by ~ 60% when compared with nondiabetics. Several key factors such as obesity (body-mass index (BMI) ≥30 kg/m^2^) and associated metabolic abnormalities, sedentary lifestyle, genetic, and environmental factors all contribute towards the development of T2D [32,33,34,35,36]. Both the first and second phases of insulin release are disturbed in T2D. A recent study demonstrated that in T2D human islets, there is a significant reduction in docked granules. Proteins associated with granule docking are downregulated in T2D, and their restoration improves granule docking [37]. There are several factors that are involved in the activation of GTPases that are dysfunctional in pancreatic β-cells from T2D individuals.

Type 1 diabetes (T1D) etiology is also now thought to begin with β-cell dysfunction, progressing to β-cell demise as a result of autoimmune attack. In the majority of patients (70–90%), the loss of β-cells is the consequence of T1D-related autoimmunity (with concomitant increase in the formation of T1D-associated autoantibodies). In a smaller subset of patients, no immune responses or autoantibodies are detected, and the cause of β-cell destruction is unknown (idiopathic T1D). This type has a strong genetic component [38]. Pathological hyper- or hypo-activation of key GTPase impairs β-cell function, especially under peripheral insulin resistance-induced stress (T2D) and oxidative stress due to pro-inflammatory cytokines (T1D). As such, targeting aspects of these signaling pathways may hold therapeutic potential for preventing β-cell failure as a means to halt the progression of T2D and T1D. (Please see the following section below).

## 4. Small Monomeric GTPases

**Figure 2:**
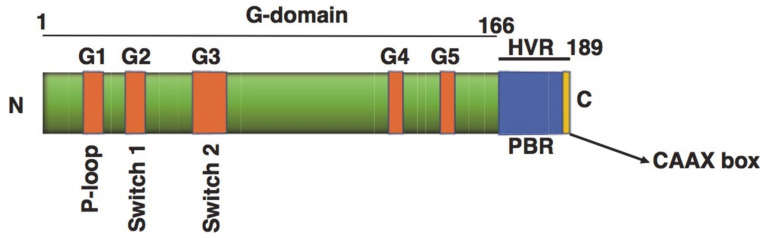
Structures of small GTPases. G boxes in the G domain (amino acids 1–166) are highlighted with orange boxes. The hypervariable (HVR) region, including a polybasic region (PBR) and a CAAX motif (166–189), is also highlighted. The CAAX box is the location in which post-translational modifications occur. The G domain consists of a six-stranded β-sheet and five α-helices, and has conserved sequence motifs G1 to G5. The G1 motif is also called the P-loop and is found in many nucleotide binding proteins, where it recognizes the β-phosphate and a Mg2^+^ ion of target nucleotides. The G2 motif (Switch 1) (Thr) makes contact with the γ-phosphate and the Mg^2+^ ion. The G3 motif (Switch 2) is responsible for GTP hydrolysis. The G4 and G5 motifs make specific contact with the guanine base to distinguish guanine from other nucleotides, Toma-Fukai et al., 2019 [46].

To date, 167 small GTPases have been identified in humans [39,40]. The GTP binding domain of these small GTPases is subdivided into five relatively conserved motifs: G1–G5 (Figure 2). The G1 motif, located between the B1 strand and the A1 helix, is responsible for binding the α and β phosphate of GTP or GDP. The G2 loop connects the A1 helix and the B2 strand, and contains a conserved threonine residue responsible for Mg^2+^ binding. The G3 motif (II) is a γ-phosphate binding region. The G4 motif (III) contains lysine and aspartic acid residues which interact directly with the guanine nucleotide. Finally, the G5 motif (IV) makes indirect associations with the guanine nucleotide [41]. The GTPases also share conserved sequences [42] at the G-box: G1, GXXXXGKS/T; G2, T; G3, DXXGQ/H/T; G4, T/NKXD; and G5, C/SAK/L/T [43]. The small GTPases can be divided into five families according to sequence similarity and function; Ras, Rho, Rab, Arf, and Ran [44]. Members of the Ras family are considered signaling hubs that modulate effector molecules to translate extracellular cues into active processes, such as secretion, cell proliferation, differentiation, morphology, and apoptosis [45].

Islet β-cells express the Rho family of small GTPases which includes RhoA, RhoB, Rac1, and Cdc42 [47,48,49,50,51,52,53,54,55,56,57]. The Rab family of small GTPases includes Rab27a, Rab3a, Rab2a, and Rab37, which regulate insulin granule formation, movement, fusion, and trafficking [58,59]. The Arf family of small GTPases includes Arf6, which is also implicated in insulin granule trafficking [60,61] (Table 1). Ran is the only member of the Ran family and is involved in nuclear transport [62].

### 4.1. Small GTPase Regulation in β-Cells

**Figure 3:**
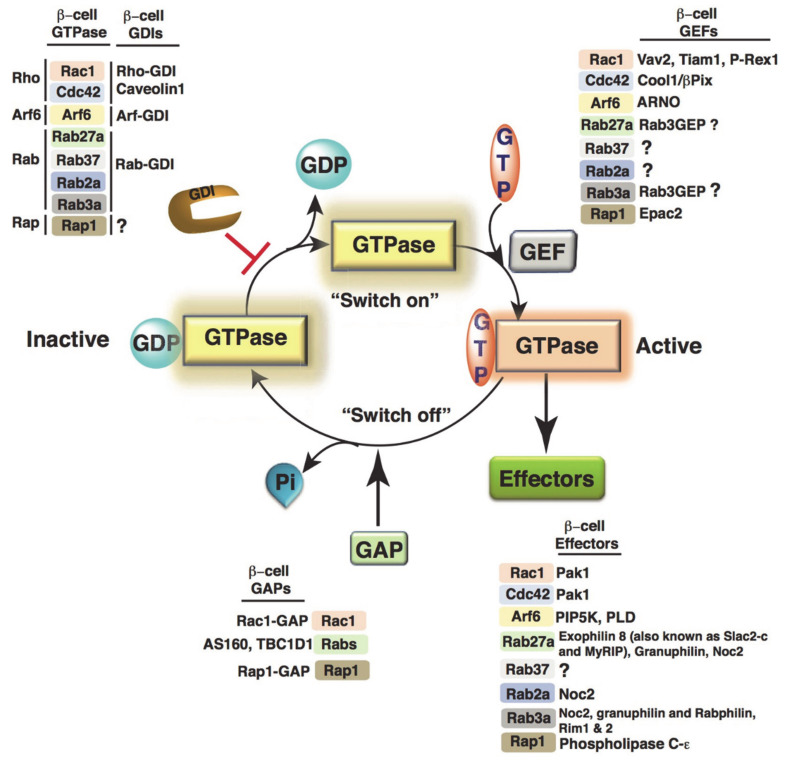
Molecular switch mechanism of GTPases. In resting cells, GTPases are bound to an inhibitory protein GDP dissociation inhibitor (GDI) in an inactive GDP-bound form. Upon stimulation, guanine nucleotide exchange factors (GEFs) facilitate the conversion of inactive GTPases to the active GTP-bound form. The active GTPase then interacts with the effector proteins to propagate the downstream signals. GTPase-activating proteins (GAPs) stimulate GTP hydrolysis from the active to inactive GTPase. The individual GEFs, GAPs, and GDIs relevant to islet β-cell signaling are listed with each GTPase.

GTPases are called molecular switches due to their regulated GDP/GTP exchange activity that evokes, or “switches on”, key signaling events in response to select stimuli (Figure 3). GTPases display high-affinity binding for GDP in the inactive state and are activated when bound to GTP. GTPases possess low intrinsic GTP hydrolysis and GDP/GTP exchange activities; the exchange of GDP/GTP is overseen by two classes of regulatory proteins. First, guanine nucleotide exchange factors (GEFs) facilitate the exchange of GDP for GTP [72]. Additionally, GTPase-activating proteins (GAPs) increase the intrinsic GTPase activity to stimulate the formation of the inactive GTPase-GDP [73]. GTPases within a family share diverse GAPs and GEFs. Although GTPases in different families depend on structurally distinct GAPs and GEFs, the mechanism by which they promote GTPase cycles remains the same. The GTP and GDP-bound states of GTPases have similar conformations, albeit with notable differences in the switch I (e.g., Ras amino acid residues 30–38) and switch II (amino acid residues 59–67) regions. Furthermore, GTPases in their GTP-bound active state possess a high affinity toward effector proteins [74,75]. The activity status of the GTPase is “sensed” by either the regulatory or the effector proteins via the conformational changes in the switch I and II domains.

### 4.2. Post-Translational Modification of Small GTPases in β-Cells

**Figure 4:**
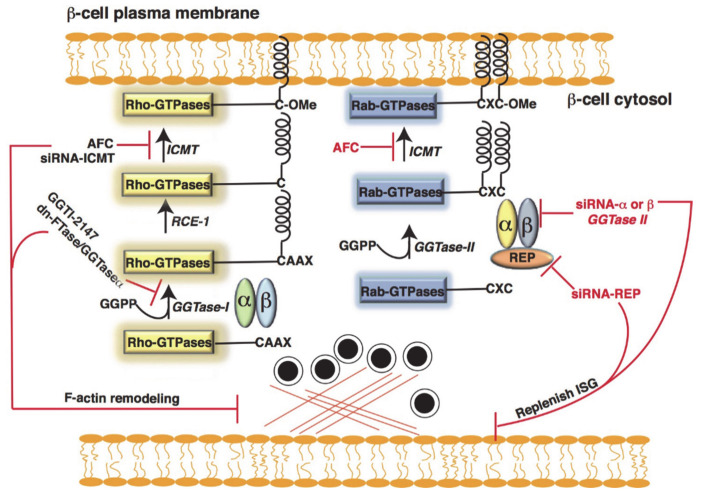
Post-translational modification of Rac1/Cdc42 and Rab-GTPases. The protein geranylgeranyl transferase-I (GGTase-I) transfers a geranylgeranyl group to proteins containing a C-terminal CAAX motif (where, C- cysteine, A- an aliphatic amino acid, and X- any amino acids). In contrast, Rab-GGTase does not recognize the CAAX motif, but requires an adaptor protein Rab escort protein (REP) to exert its function. REP recruits newly synthesized Rab-GTPases, and then presents them to the Rab-GGTase (GGTase-II). Once the geranylgeranyl group is inserted, as in the case of Rac1/Cdc42, the three terminal amino acids are removed by a Ras converting enzyme 1 (RCE-1). Furthermore, a methyl group is inserted onto the carboxylate anion of the prenylated cysteine via an ester linkage in the presence of S-adenosyl methionine by an enzyme protein-S-isoprenylcysteine *O*-methyltransferase (ICMT).

The small GTPases, which contain a CAAX box in their carboxyl terminus region, can undergo series of post-translational modifications that make them hydrophobic and facilitate their interaction with proteins, such as the Rho protein GDP dissociation inhibitor (Rho-GDI), or membranes [76,77]. These modifications include attachment of either farnesyl pyrophosphate (FPP; e.g., H-Ras) or geranylgeranyl pyrophosphate (GGPP; e.g., Rac1, Cdc42, and Rap1) to the cysteine residue of the CAAX motif by farnesylation or geranylgeranylation, respectively [77]. Farnesylation occurs at the CAAX motif when the sequence ends in any amino acid other than leucine and is catalyzed by farnesyl transferase (FTase) [78]. In contrast, geranylgeranylation occurs when the CAAX sequence ends in leucine and is catalyzed by geranylgeranyl transferase-I (GGTase-I) [79]. Various subunits of FTase and GGTase have been identified in islet β-cells [80]. The enzyme geranylgeranyl transferase-II (GGTase-II), also known as Rab geranylgeranyl transferase (Rab-GGTase) [81], has been identified in islet β-cells [82]. Rab-GGTase fails to recognize the CAAX box and requires an adaptor protein, the Rab escort protein (REP) [82], to exert its function. REP recruits newly synthesized Rab-GTPases and presents them to the Rab-GGTase [83]. Once a ternary complex is formed (α and β subunits of Rab-GGTase, REP, and the incoming Rab-GTPase), two geranylgeranyl groups are transferred onto the C terminus of Rab-GTPase [81,84], and REP-bound geranylgeranylated Rab is escorted to the respective target membrane (Figure 4).

Extensive studies have been conducted in islet β-cells, wherein post-translational lipid modification (farnesylation and geranylgeranylation) was reduced using pharmacological agents such as lovastatin [85,86] (a general inhibitor of the mevalonic acid biosynthesis pathway), structure-specific inhibitors, such as 3-allyl/vinyl-farnesols and 3-allyl/vinyl geranylgeraniols [87], and overexpression of dominant-negative FTase/GGTase-I, a common subunit of FTase or GGTase [63]. These studies demonstrated that inhibiting this lipid modification caused accumulation of the small GTPases in the soluble compartment rather than at the membrane, thereby reducing the interaction with effector proteins and attenuating GSIS.

#### Metabolic Dysfunction and Defective Post-Translational Modification of GTPase in Islet β-Cells

Increasing evidences demonstrate that the statin cholesterol-lowering drugs, which inhibit 3-hydroxy-3-methylglutaryl-CoA (HMG-CoA) reductase, reduce atherosclerotic cardiovascular burden [88], but are associated with increased incidence of new-onset T2D in a dose-dependent manner [89,90,91,92]. Several clinical studies demonstrated that statin therapy increased the incidence of diabetes, including Justification for the Use of Statins in Prevention: An Intervention Trial Evaluating Rosuvastatin (JUPITER) [93], in which a large, randomized, placebo-controlled, primary prevention trial demonstrated that rosuvastatin (20 mg/day) decreased the incidence of adverse vascular events in the rosuvastatin group by 44%, whereas saw an increase of 26% incidence of diabetes in the rosuvastatin group. Similarly, the prospective study of pravastatin in the elderly at risk found a 32% higher incidence of diabetes with pravastatin therapy [94]. A recent meta-analysis by Sattar et al. [90] included 13 randomized, placebo-controlled, and standard care controlled trials (including JUPITER and WOSCOP) with 91,140 participants. This meta-analysis revealed a 9% increase in the risk of diabetes incidence with little heterogeneity between trials, and the risk was more significant in elderly patients. In Diabetes Prevention Program Outcomes Study [95], the population at high risk for diabetes had significantly higher rates of diabetes with statin therapy. Taken together, statins may ‘reveal’ diabetes in individuals at high risk, depending on ethnicity, and results in a modest increase in diabetes risk.

Statins prevent de novo cholesterol biosynthesis and isoprenoid intermediates, such as farnesyl pyrophosphate (FPP) and geranylgeranyl pyrophosphate (GGPP) [96]. Several proteins, including Cdc42, Rac1, and Rho, undergo prenylation in islet β-cells by GGTase-I, whereas GGTase-II (also referred to as the Rab-GGTase) prenylates the Rab subfamily of proteins. Studies in β-cells have demonstrated that inhibition of GTPase prenylation causes defective GSIS due to defects in processes such as cytoskeletal remodeling, insulin secretory granule trafficking and fusion events [82,96,97]. For example, β-cell-specific deletion of geranylgeranyl pyrophosphate synthase (GGPPS), a protein prenylation enzyme, resulted in reduced GSIS coincident with fewer than normal insulin granules trafficking to the PM [98]. Furthermore, GGPP administration restored GSIS in GGPPS-null islets. In addition, GGPPS expression in db/db mice is increased during the insulin compensatory period of T2D development, followed by a decrease during the β-cell dysfunction phase [98]. Thus, statins might affect β-cell function by disturbing protein prenylation.

## 5. Rho-GTPases in Islet β-Cells

### 5.1. Cdc42

Cdc42 plays an important proximal role in regulating islet β-cell function [47,48,49,65,99]. Studies using GTPγS, a non-hydrolyzable G-protein-activating analog of GTP, demonstrated that the formation of Cdc42-GTP (active conformation) is required for Cdc42 to translocate to the PM [66]. Post-translational prenylation is key for the association of Cdc42 with the inhibitory protein Rho-GDI in β-cells; exposure to a prenylation inhibitor prevents the association of Cdc42 with Rho-GDI [50]. Second-phase insulin release requires mobilization of insulin granules located deep inside the intracellular storage pools toward the PM and involves glucose-induced remodeling of the actin cytoskeleton [100,101,102], a process involving the localized and transient conversion of filamentous actin to globular actin (F-actin to G-actin) to permit granule movement through the network.

Cdc42 activation in response to glucose stimulation was found to occur early during the first-phase of GSIS (~2–3 min) [65]. The activated form (Cdc42-GTP) localizes to the PM [48], after which it cycles back to Cdc42-GDP due to the glucosylation of Cdc42 [47]. The timing of Cdc42 activation/deactivation correlated with the visualization of F-actin depolymerization and repolymerization, consistent with the hypothesis that GSIS is regulated by Cdc42 cycling. Furthermore, expression of the Q61L constitutively-active mutant of Cdc42 yielded blunted cortical actin depolymerization that correspondingly inhibited GSIS [47]. Cdc42 was found to co-localize with VAMP2-containing insulin secretory granules in pancreatic β-cells and to translocate to the PM when stimulated with glucose. Cdc42-VAMP2 complexes in the PM were also found to associate with syntaxin 1A, further promoting insulin granule fusion and release [48].

Surprisingly, the caveolar protein Caveolin1 (Cav-1) was also identified as GDI for Cdc42, specifically for the pool of Cdc42 which localizes to the insulin granules—in this location, Cav-1 forms a heterotrimeric complex with Cdc42 and VAMP2 [49]. Cav-1 contains a Ras binding domain, which mediates a direct interaction with Cdc42 [49]. Cav-1 dissociates from the Cdc42-VAMP2 complex upon glucose stimulation at the same time that Cdc42 associates with β-Pix [103], a β-cell GEF for Cdc42. Depletion of Cav-1 from isolated islets and clonal MIN6 β-cells was shown to elevate basal insulin release and attenuate GSIS, simulating the β-cell dysfunction associated with T2D [49]. Consistent with the role of Cav-1 as a GDI for Cdc42, with Cdc42 activation in the β-cell being upstream of ERK1/2, Cav-1 deficient β-cells showed increased activities of ERK1/2; this triggered events related to proliferation, such as downregulated cell cycle inhibitors (e.g., FOXO1 and GSK3β) and upregulated expression of Cyclin D2 and Cyclin D3 [104,105]. Intriguingly, whole body Cav-1 knockout mice fed a high fat diet showed less β-cell apoptosis than control mice [106], whereas Cav-1 overexpression in MIN6 β-cells exacerbated the palmitate-induced increase in β-cell apoptosis [104]. These findings add to the growing body of knowledge suggesting that β-cells toggle between function (GSIS) and proliferative states.

In further support of a key role for Cdc42 in the β-cell in regard to whole body glucose homeostasis, mice lacking the Cdc42 gene in pancreatic β-cells (Rip-CDC42cKO) were shown to display glucose intolerance and decreased GSIS, without alterations to islet morphology [107]. Furthermore, Rip-CDC42cKO mice showed reduced signaling via the ERK1/2-NeuroD1 pathway and attenuated insulin expression. Together, these data supported the earlier in vitro findings demonstrating Cdc42 to be an essential modulator of GSIS in pancreatic β-cells [107]. An additional study has demonstrated that miR-29a targets the Cdc42 mRNA 3′-UTR and negatively regulates Cdc42 and the downstream molecule β-catenin, inhibiting proliferation and insulin secretion in the clonal MIN6 β-cell line [108]. Furthermore, human islet perifusion assays further demonstrated that overexpression of miR-29 inhibited GSIS [109]. Beta cell-specific transgenic miR-29a/b/c overexpressing mice have an increased susceptibility for high-fat diet-induced glucose intolerance compared to control mice [109]. Future studies using an inducible β-cell specific miR-29a overexpressing mouse model will be important to assess this potential linkage of miR-29a and Cdc42 in the context of biphasic GSIS and β-cell viability.

Defects in lipid homeostasis are closely associated with insulin secretion defects, a key feature of T2D. In a recent study, depletion of ABCA12, a lipid transporter protein in mouse β-cells, resulted in impaired GSIS, islet inflammation, and β-cell death [110]. In particular, the impaired GSIS was linked to defects in the biogenesis and fusion of insulin secretory granules that were associated with dysregulated Cdc42-induced actin cytoskeletal remodeling [110]. Taken together, impairments in small GTPases within islet β-cells clearly contribute toward the etiology of T2D [97,98,110,111].

### 5.2. Rac1

In β-cells, Cdc42 activation and cycling leads to the activation of Rac1, and knockdown of Cdc42 reduces the activation of Rac1 [65]. The importance of Rac1 activation and cycling in islet β-cells was first demonstrated using *Clostridium difficile* toxins A and B, which irreversibly monoglucosylate and inactivate Rac1 [53]. Using an inactive mutant of Rac1 (N17Rac1), Li et al. [54] demonstrated that activation of Rac1 (Rac1-GTP) is required for its translocation to the inner leaflet of the PM, and this translocation occurs immediately before the disappearance of F-actin structures, wherein this F-actin depolymerization is known to underlie insulin granule mobilization to the cell surface to support the second phase of GSIS.

These observations were later corroborated using β-cell-specific Rac1 knockout mice [112]. The βRac1^−/−^ mice were developed by crossing Rac1^flox/flox^ mice, which harbor a modified endogenous Rac1 gene in which exon1 is flanked by loxP sites, with those that express the Cre recombinase gene under the control of the rat insulin-2 gene. βRac1^−/−^ mice developed glucose intolerance and hypoinsulinemia, despite normal islet density and β-cell mass [112]. While a line of β-cell-specific N17Rac1 (RIP-RacN17) mice has also been generated and phenotyped, neither glucose tolerance nor insulinemia levels have been evaluated [113]. To determine whether Rac1 activation is required in β-cells in vivo, it will be interesting to compare the phenotypes of mice with N17Rac1 versus WT Rac1 expressed in the context of the β-cell-specific Rac1 KO background.

Additional evidence supporting the importance of Rac1 activation in the β-cell comes from pharmacologic studies using NSC23766 and Ehop-016 [64,114]. NSC23766, a chemical compound that inhibits Rac1-GEF interaction [115] and prevents glucose-induced Rac1 activation, inhibits Rac1 trafficking to the cell surface, correlating with the loss of GSIS, in INS-1 832/13 cells and rat islets [64]. β-cell-specific knockdown of the Rac1-GEF T-lymphoma and invasive metastatic protein (Tiam1) produces a similar phenotype [64]. In addition to Tiam1, Vav2 has been identified as a GEF in the regulation of Rac1-mediated GSIS and actin remodeling; this was confirmed using a small molecule inhibitor of the Vav2-Rac1 interaction: Ehop-016 [114]. Vav2-GEF activity can be regulated by tyrosine phosphorylation [116,117]. Several Src family tyrosine kinases, such as Lck, Fyn [118,119], and Syks (Syk and Zap70) [119,120], as well as receptor tyrosine kinases [121,122,123], have been implicated as mediators of Vav2 tyrosine phosphorylation. Recently, the tyrosine kinase Yes (a Src family kinase) has been reported to regulate Cdc42 activation in a glucose-dependent manner in pancreatic β-cells [124]. It remains untested whether Cdc42 and Rac1 are both activated by Yes kinase; such a hypothesis is intriguing, although it is important to note that activation of Cdc42 and Rac1 occur as temporally distinct events in β-cells [47,48,65].

### 5.3. Rho-GDI

Cdc42 and Rac1 share an inhibitory GDI regulator, Rho-GDI, which sequesters Rho-GTPases to prevent dissociation of GDP and prevent translocation to the membrane for GEF interaction and activation. Overexpression of the WT Rho-GDI significantly attenuated glucose-induced, but not KCl- or Mastoparan (peptide toxin from wasp venom and global activator of GTPases)-induced, insulin secretion. Conversely, siRNA-mediated knockdown of endogenous Rho-GDI increased GSIS [125,126]. Together, these studies suggested an inhibitory role for Rho-GDI in GSIS.

Dissociation of Rho-GTPases from Rho-GDI is a vital step in the activation of GTPases. Studies in β-cells have demonstrated a temporal and spatial difference between Cdc42 and Rac1 interactions with Rho-GDI [65,125]. Co-immunoprecipitation studies have revealed that Rho-GDI-Cdc42 complexes in β-cells dissociate within 3 min after stimulation with glucose, correlating with the timing of Rho-GDI tyrosine (Tyr-156) phosphorylation [65]. Glucose-induced disruption of Rho-GDI-Rac1 complexes occurred around 15 min, an event coupled with Rac1 activation [125]. Interactions between Rho-GTPases and Rho-GDIs can be regulated by post-translational phosphorylation, sumoylation, acetylation, and oxidation. Phosphorylation of Rho-GDIs decreases their affinity for Rho-GTPases, thereby promoting the release of Rho-GTPases and their subsequent activation by Rho-GEFs [127,128]. For example, phosphorylation of Rho-GDI1 by protein kinases, such as Src, PKCα, p21 activated kinase 1 (PAK1), and FER, facilitate the release of specific Rho-GTPases and their subsequent spatiotemporal activation [129,130,131,132,133]. Rho-GDI-Rac1 dissociation was blocked by the Rho-GDI mutations Y156F and S101A/S174A, which block phosphorylation, suggesting a spatiotemporal regulation of Rho-GDI-Rac1/Cdc42 disruption and Cdc42/Rac1 activation in β-cells [65,125]. Src phosphorylates Rho-GDI1 at Tyr156 to promote translocation of Rho-GDI to the PM and local activation of RhoA, Rac1, and Cdc42 [129]. Yes kinase is implicated in phosphorylation of Cav-1 in β-cells, wherein Cav-1 acts as a GDI for Cdc42 in the insulin secretory granules [124]. It will be important to clarify if Yes kinase also disrupts the Cdc42-Rho-GDI complex in β-cells. Rho-GDI1 phosphorylation at Ser101 and Ser174 by PAK1 promotes dissociation and activation of Rac1, but not RhoA [132].

PAK1 activation by Rac1 and Cdc42 regulates insulin release; therefore, there may exist positive feedback between PAK1 activation and Rho-GDI phosphorylation for Rac1 signaling. However, phosphorylation of Rho-GDIs does not always induce the disruption of the GTPase-GDI complex. Cyclic AMP-dependent protein kinase A (PKA) phosphorylates Rho-GDI1 at Ser174 and RhoA at Ser188, which increases the affinity of Rho-GDI1 to RhoA, thereby inhibiting RhoA signaling [134,135]. Protein phosphatase 1B dephosphorylates Rho-GDI1 to decrease Rho-GDI1 interaction with 14-3-3ι and activate Rho-GTPases [136]. Therefore, reversible phosphorylation of Rho-GDIs by a kinase/phosphatase could be a critical mechanism for precisely regulating the spatiotemporal activation of Rho-GTPases [137].

**Figure 5:**
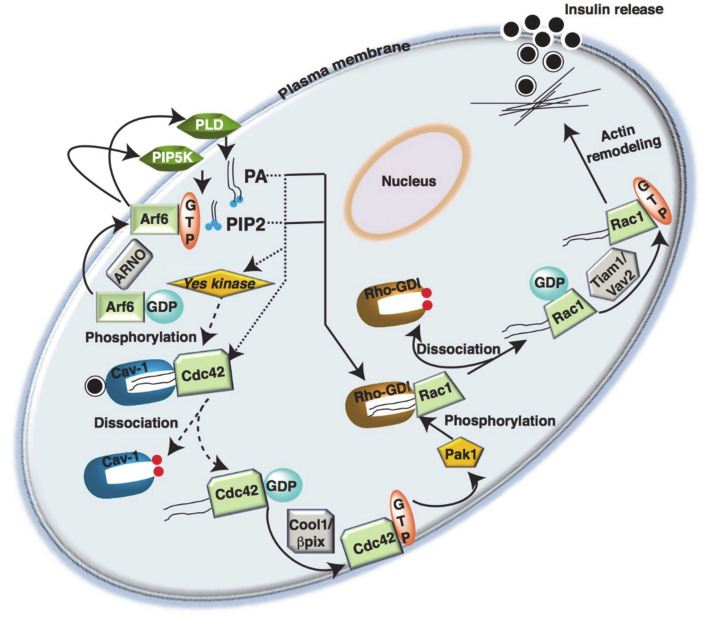
Cross-talk between Arf6 and Rho-GTPases in islet β-cell insulin secretion. Glucose metabolism leads to activation of Arf6, which initiates the activation of membrane associated phospholipase D (PLD) and phosphatidylinositol-4-phosphate 5-kinase (PIP5K) to generate fusogenic lipids such as phosphatidylinositol 4,5-bisphosphate (PIP2) and phosphatidic acids (PA). The lipids generated facilitate the dissociation of Cdc42 from Cdc42/Caveolin-1 complex present in the insulin containing vesicle, when the caveolin-1 is phosphorylated by a Yes kinase, and subsequent activation by Cool/β-pix (GEF). The activated Cdc42 activates Pak1, which phosphorylates and activates Rac1/GDI. This leads to exchange of GDP/GTP to initiate cytoskeletal rearrangement for insulin granule mobilization and release.

In addition, lipid kinases such Phospholipase D (PLD) or Phosphatidylinositol-4-phosphate 5-kinase (PIP5K) are targets of Rho-GTPases [138], as phospholipids (phosphatidylinositol 4,5-bisphosphate (PIP2), phosphatidic acid (PA)) may also act as Rac/Cdc42-Rho-GDI complex disruption agents [139,140,141] (Figure 5). The presence of acidic phospholipids such as PIP2 and PA can promote the release of Rho-GTPases from Rho-GDIs [142,143]. Phospholipids mediate a partial opening of the complex that exposes the GTPases to GEFs [144]. A possible mechanism could involve competition of the active lipids for Rho binding sites on the GDI protein. Analysis of the primary structure of human Rho-GDI reveals a single hydrophobic region (amino acids 50–80) and this region can interact with the hydrophobic isoprenyl group at the C terminus of Rho-GTPases, consistent with the known requirement of this modification for the binding of Rho family proteins to Rho-GDI [139].

#### Rac1 and Oxidative Stress in T2D Islet β-Cells

β-cells are highly susceptible to oxidative stress due to their relatively low levels of antioxidant enzymes, such as catalase and glutathione peroxidase [145]. There are several sources of reactive oxygen species (ROS) production in β-cells: nonenzymatic glycosylation [146], the electron transport chain in mitochondria [147], and the hexosamine pathway [148]. Recently, β-cells have been shown to express NADPH oxidase (NOX2) [149], which is predominantly localized at the PM. Glucose, palmitate, and pro-inflammatory cytokines modulate expression via post-translational modification of the p47*^phox^* NOX2 subunit; these cytokines also modulate NOX2 activity in rat pancreatic islets and clonal β-cells [150]. The NOX macromolecular complex consists of several subunits, including two membrane subunits (gp91*^phox^* and p22*^phox^*), three cytosolic subunits (p47*^phox^*, p67*^phox^*, and p40*^phox^*), and the small GTPase Rac1 [151]. It has also been shown that activation of Rac1 is vital for the holoenzyme assembly and activation of NOX [152]. Indeed, protein levels of active Rac1, NOX activity, ROS generation, Jun NH2-terminal kinase (JNK) 1/2 phosphorylation, and caspase-3 activity were significantly higher in Zucker diabetic fatty rat (ZDF) islets and T2D human islets [153,154]. Inhibition of Rac1 activation significantly attenuates NOX2-driven p38MAPK phosphorylation, implicating a regulatory role for Rac1 in promoting NOX2-p38MAPK signaling in β-cell [155]. In T1D, Rac1, a part of NOX2, is involved in the generation of ROS under the duress of cytokine stress. Furthermore, NSC23766, a small inhibitor of Rac1-Tiam1 signaling module prevented the spontaneous on set of diabetic phenotype in NOD mice. The prevention of diabetes is due to significant inhibition of Rac1 expression and activity, which is otherwise significantly elevated in NOD mice without NSC23766 treatment [156]. The mechanism by which Rac1 regulates two seemingly opposing phenomena in the β-cell, i.e., promoting GSIS and NOX2 activation, is still under scrutiny. A plausible explanation could be that inhibition of geranylgeranylation constitutively activates Rac1 [97,98,157].

### 5.4. Arf

Arf belongs to the Ras superfamily [44]. Similar to Cdc42 and Rac1, Arf6 associates with the inner leaflet of the PM in a GTP-dependent manner, and removal of GTP is needed to dissociate Arf6 from the PM [158]. At the PM, Arf6 directly activates membrane lipid-modifying enzymes, such as PIPK5 [159] and PLD [160], which leads to the production of PIP2 and PA, respectively [161]. The generation of PIP2 at the PM is critical for Arf6 mediated endocytosis, endosomal recycling, PM remodeling, and actin polymerization [161]. In β-cells, expression of a dominant-inhibitory Arf6 mutant, Arf6(T27N), impaired GSIS in the pancreatic MIN6 β-cell line [162]. In response to membrane depolarization, MIN6 cells expressing Arf6(T27N) showed loss of second-phase insulin secretion, with normal first phase secretion, consistent with the roles for small GTPase cycling in the actin remodeling-based second phase of GSIS. One caveat in this study is that the authors derived their conclusions from MIN6 clonal β-cells, and did not confirm the findings in primary islet β-cells. Although the basal levels of PIP2, derived from PIPK5, are sufficient to keep a pool of insulin secretory granules primed and ready for release during the first phase of secretion [162], Arf6 plays a crucial role in the priming of insulin secretory granules to support second-phase insulin secretion.

Furthermore, the Arf nucleotide binding site opener (ARNO) was identified as a GEF for Arf6 in β-cells [163] and overexpression of ARNO inactive mutants and secinH3, a selective pharmacological inhibitor of ARNO/Arf6, inhibited Arf6, Cdc42, and Rac1 activation and GSIS in INS-1 832/13 cells and rodent islets [163], suggesting that ARNO/Arf6 might be upstream of Cdc42 and Rac1 activation. These studies could also be recapitulated using RNA interference (RNAi) experiments, where the authors demonstrated that reducing ARNO expression completely suppressed glucose-induced activation of Rac1. Multiple signaling pathways are postulated to emanate from Arf6 activation in β-cells, including the regulation of mTOR and p70S6K, based upon studies performed in MIN6N8 cell line [164,165]. High glucose induced the binding of Arf6 to PLD1, a protein that catalyzes the hydrolysis of phosphatidylcholine to generate PA and choline [166]. PLD1 has been implicated in glucose-induced mTOR and p70S6K signaling in the β-cell, tentatively placing it downstream of Arf6, although this mechanistic placement awaits further experimental evidence using specific Arf6 and PLD1 genetic and pharmacologic tools.

### 5.5. Rab-GTPases

Rab-GTPases, the largest family of small GTPases, regulate intracellular membrane traffic [167,168]. Different Rab-GTPases localize to separate membrane compartments, thereby controlling the specificity and directionality of membrane trafficking, serving as membrane identity markers [169,170,171]. Rab3A was the first Rab-GTPase identified on insulin-containing secretory granules. Using mouse Rab3a knockout [70,172], and overexpression models [173,174,175,176], researchers demonstrated that Rab3a is required for normal insulin secretion and the control of plasma glucose levels. Rab27A, a close evolutionary relative of Rab3, is also associated with insulin secretory granules [70,177,178]. In studies using Rab27a-deficient mice [70,170,171] or Rab27a overexpressing mice [67,179], it is clear that Rab27a regulates insulin release. Ashen mice carry a point mutation resulting in excision of Rab27a, and Ashen β-cells show disrupted refilling of the readily releasable pool of insulin secretory granules in response to stimulatory glucose, potentially placing Rab27a as a regulator of second phase GSIS. In contrast, Rab3a^−/−^ mice exhibited normal refilling of the readily releasable pool, distinguishing the role of Rab3a from that of Rab27a [175,176,180].

The Rab27a effector, granuphilin, localizes to insulin granules [181] at the PM. Granuphilin associates with the t-SNARE protein syntaxin 1A, as demonstrated by co-immunoprecipitation and in vitro binding assays [67]. Overexpression of a granuphilin mutant that impairs granuphilin binding to Rab27a or syntaxin 1A causes dysfunctional GSIS [67,180,181], supporting the concept that granuphilin plays a role in connecting specific pools of insulin secretory granules to specific SNARE proteins at the PM.

Two other types of Rab27a effectors, Synaptotagmin-like proteins (Slps) and the related Slac2 proteins, which are Slps lacking C2 domains; each harbor a unique amino-terminal domain that confers binding to Rab27 [182,183]. One in particular, Slac2c/MyRIP (Myosin VIIA and Rab Interacting Protein), is also associated with the secretory granules of β-cells [179]. Slac2c/MyRIP knockdown impairs GSIS. β-cells express Myosin-Va, a potential binding partner of Slac2c/MyRIP [179]. Indeed, overexpression of just the actin-binding domain of Slac2c/MyRIP suppressed insulin granule exocytosis, suggesting Rab27a and Slac2c/MyRIP co-regulate the interaction of secretory granules with the cortical actin cytoskeleton and participate in insulin exocytosis.

Beyond Rab27a and Rab3, two other Rab-GTPases are implicated in β-cells: Rab2a and Rab37. While Rab27a regulates the late stage of the secretory process by facilitating granule recruitment to the fusion site, Rab2a functions in the early stage of insulin granule biogenesis; however, both bind to the same Rab effector protein, Noc2 (Nucleolar complex protein 2 also known as RPH3AL). The GTP-dependent ternary Rab2a-Noc2-Rab27a complex specifically localizes on perinuclear immature granules, whereas the binary Noc2-Rab27a complex localizes on mature granules at the PM. Noc2 mutants defective in binding to Rab2a or Rab27a were found to inhibit GSIS, while knockdown of Rab2a or Noc2 impairs the maturation of insulin secretory granules [69]. Rab37 is essential for GSIS, regulating the number of granules in close proximity to the PM. Interestingly, Rab37 does not bind to any of the effectors of Rab3a or Rab27a, suggesting the effectors linking Rab37-GTP and GSIS remain to be found [68]. Nevertheless, the Rab-GAP TBC1D1 has been implicated as a required Rab cycling protein given that TBC1D1-knockout β-cells harbor increases in insulin granule density and number of docked granules, supporting the observed elevated first- and second-phase insulin release [184].

#### Arf6 and Rab27a Couple Exocytosis and Endocytosis

Exocytosis-endocytosis coupling in the pancreatic β-cell was first demonstrated by Orci et al. [185]; this coupling is essential to maintain PM homeostasis, as the extra portion of the lipid membrane added during the exocytosis of insulin granules is cleaved to maintain the original structure of the PM and cellular circumference/size [186]. GSIS is coupled to the budding/endocytosis of PM material [185]. Conventional endocytosis, as reported in other cell types [187,188,189], involves recruitment of clathrin, which, along with a host of adaptor proteins, initiates an inward curvature of the PM. Then, dynamin GTPase facilitates the constriction and excision of the endocytotic vesicle from the PM [189]. In pancreatic β-cells, glucose also promotes phosphatidylinositol (3,4,5)-trisphosphate (PIP3) generation through phosphoinositide 3-kinase (PI3K), and further recruits ARNO and EPI64 (Rab27a-GAP) to the inner leaflet of the PM, where ARNO promotes clathrin assembly via Arf6 and synchronizes early endocytosis [190,191]. On the other hand, Rab27a facilitates exocytosis [179,192,193] (see Section 5.4). Thus, these results indicate that the glucose-induced activation of PI3K plays a pivotal role in exocytosis-endocytosis coupling, and that ARNO and EPI64 regulate endocytosis at distinct stages [179,190,191,192,193].

### 5.6. Rap-GTPases

Rap1 GTPase and its GEF, Epac2, are the major forms of their respective families in islet β-cells [194,195,196]. Rap1 activity is maintained by the PKA-mediated phosphorylation of Rap1-GAP, thus disallowing Rap-GAP to hydrolyze GTP from Rap1 [197,198]. A PKA-independent pathway involves the Rap1-GEF, Epac2. [199]. In mouse islets, a PKA inhibitor (H89) and Epac2 antisense oligonucleotide (ASOs) each partially inhibited glucagon-like peptide-1 (GLP-1)-induced potentiation of GSIS, while the combined treatment had an additive effect [200]. Rap1 is also known to enhance islet cell proliferation exclusively via mammalian target of rapamycin complex 1 [201].

Rap1 signaling through Epac2 facilitates the insulin release enhancing effects of GLP-1. GLP-1 binds to its cognate GLP-1 receptor (GLP1R), a G-protein coupled receptor found largely on the surface of pancreatic β-cells (as well as α- and δ-cells) [202,203], which increases the intracellular cAMP levels by activating transmembrane associated adenylate cyclases (TMACs) [204] and potentiating insulin secretion. Indeed, GLP1R agonists, such as GLP-1, are used clinically to increase insulin release from residual endogenous β-cells of the T2D islet [205,206,207]. Most recently, an Epac2 activator was shown to activate Cdc42 [111], which is potentially another instance where small GTPase regulatory proteins are used by multiple GTPases in the β-cell. Further studies are required to delineate the multiple effects of Epac2 in β-cells. Nevertheless, current data suggest that most GLP1R signaling is mediated by PKA, Epac2-Rap1, and Cdc42 in β-cells. Additionally, activation of Rap1 binds to and activates phospholipase C-ε. PLC-ε initiates the generation of diacylglycerol (DAG) and inositol trisphosphate (IP3). DAG and Ca^2+^ activate protein kinase C (PKC), and PKC phosphorylates secretory granule-associated proteins, thereby increasing insulin granule exocytosis [208,209].

## 6. Identification of GTPase Regulating Proteins as T2D Candidate Genes

T2D genome-wide association analysis meta-analysis revealed two IQ-motif-containing GAPs (IQGAP1 and IQGAP2), involved in cellular signaling, cytoskeletal organization, and GSIS [210] in the top 95^th^ percentile for association with T2D; providing evidence for IQGAP2 contribution to insulin resistance [211]. Recently, a GEF for Rac1, P-Rex1, which is activated by PIP3 via PI3K, has been shown to play a critical role in GSIS in insulin-secreting INS-1 832/13 cells [212]. Single nucleotide polymorphism analysis of the 3′ perigenic region of P-Rex1 was found to be associated with an increased risk of obesity and T2D development. In humans, the P-Rex1 locus is near a region on chromosome 20 which is associated with T2D (20q12–13.1) [213]. TBC1D4, a Rab-GAP, was recently identified in GWAS as a T2D-associated SNP in Greenlandic individuals, and is known to cause insulin resistance in skeletal muscle [214]. TBC1D4 has been identified in human and mouse pancreatic β-cells, and TBC1D4 mRNA expression was found to be downregulated in pancreatic islets from individuals with T2D [215]. Furthermore, TBC1D4 knockdown decreased GSIS from a MIN6 β-cell line [215] and human EndoC-βH1 cells [216]. The human Rho-GEF11 (ARHGEF11) R1467H variant in a German Caucasian cohort was linked to impaired glucose tolerance and increased susceptible to T2D, implicating a GTPase regulatory protein and T2D risk [217]. As such, β-cell defects in insulin secretion could be caused by dysregulated GTPase regulatory proteins.

## 7. Conclusions

In this review, we summarize the evidence for the role of different small GTPases in the islet insulin secretory process, including actin cytoskeletal rearrangement, vesicle trafficking, and vesicle fusion. We also review how these GTPases are regulated and the associated regulatory factors that play a vital role in β-cell function. Furthermore, we describe how the functional impairment of GTPases can lead to β-cell dysfunction and how GTPase signaling can revive otherwise dysfunctional β-cells. However, this research area is in its infancy, and further mechanistic in vivo studies are required, which will lead to future discoveries.

## 8. Future Directions

More research is needed to understand how GTPase cycling coordinates to facilitate second-phase insulin secretion, a process that requires actin cytoskeleton dynamic changes with signaling cues to fill the insulin secretory granule pools. It will also be important to determine the spatiotemporal regulation of Rho-GDI in sequestering active GTPases in their inactive forms and to understand how several GEFs coordinate the activities of the GTPases Rac1 and Cdc42.

Similarly, cross-talk between several Rab and Ras family GTPases in granule docking and priming, and their roles in biphasic insulin secretion, remain unresolved. New methodologies are required to study the spatiotemporal regulation of GTPases in response to the dynamic changes in the intracellular locations of Rho-GEF, Rho-GAP, and Rho-GDI. Optogenetics allows researchers to reversibly trigger signaling with spatial and temporal control in the sub-second range when combined with genetically encoded biosensors and real-time nuclear magnetic resonance (RT-NMR) experiments allow the collection of spectra for kinetic analyses of exchange or hydrolysis of GTPases. Such assays will provide new evidence to unveil the role of GTPases in control of β-cell insulin granule mobilization, tethering, docking, priming, and fusion in real time.

## Figures and Tables

**Table 1:** Location and function of GTPases in the islet β-cell.

GTPases in β-Cells	Location	Function	References
Rac1	Cytosol (GDP-loaded), PM (GTP-loaded)	Cytoskeletal rearrangement	[63,64]
Cdc42	Cytosol/Insulin-containing secretory granules (GDP-loaded), PM (GTP-loaded)	Cytoskeletal rearrangement/ Vesicle fusion	[47,48,49,65,66]
Arf6	Cytosol (GDP loaded), PM (GTP loaded)	Vesicle fusion	[50]
Rab27a	Insulin-containing secretory granules (GDP and GTP loaded)	Docking/priming	[67]
Rab37	Cytosol (GDP loaded) Insulin-containing secretory granules (GTP loaded)	Docking/priming	[68]
Rab2a	Cytosol (GDP loaded) Perinuclear immature granules (GTP loaded)	Docking/priming	[69]
Rab3a	Cytosol (GDP loaded) Insulin-containing secretory granules (GTP loaded)	Docking/priming	[70]
Rap1	Colocalized with insulin granules (GDP loaded), PM (GTP loaded)	Docking/priming	[71]

PM, plasma membrane.

## Data Availability

Not applicable.
